# Iliac artery aneurysm endoleak management in a patient with vascular Ehlers-Danlos syndrome

**DOI:** 10.1016/j.jvscit.2023.101401

**Published:** 2024-01-17

**Authors:** Mitri K. Khoury, Matthew J. Eagleton

**Affiliations:** Division of Vascular and Endovascular Surgery, Department of Surgery, Fireman Vascular Center, Massachusetts General Hospital, Boston, MA

**Keywords:** Connective tissue, Ehlers Danlos, Endoleak, Iliac aneurysm, Transgluteal

## Abstract

Endovascular repair has traditionally been avoided in patients with connective tissue disorders. We describe successful treatment of multiple endoleaks of an expanding common iliac artery aneurysm previously treated with an endograft in a patient with vascular Ehlers-Danlos syndrome. The modalities used to treat the endoleaks were transgluteal embolization of the internal iliac artery and proximal and distal extension of the prior endograft. This case demonstrates endovascular management of endoleaks in patients with vascular Ehlers-Danlos syndrome can be safe and feasible.

Ehlers-Danlos syndrome is an autosomal dominant connective tissue disorder characterized by skin elasticity, joint hypermobility, bruising, and vascular complications. There are 13 subtypes of Ehlers-Danlos syndrome but types I, III, and IV are associated with vascular complications.^1^ Type IV Ehlers-Danlos syndrome is also known as vascular Ehlers-Danlos syndrome (VEDS) and results from mutations in *COL3A1,* leading to deficiencies in the synthesis and excretion of type III procollagen. Approximately 17% to 37% of VEDS patients experience iliac pathology during their lifetime.^2^, ^3^, ^4^ We present a case of a patient treated endovascularly for an expanding common iliac artery (CIA) aneurysm due to multiple endoleaks after prior stent placement. The patient provided written informed consent for the report of her case details and imaging studies.

## Case report

**Fig 1:**
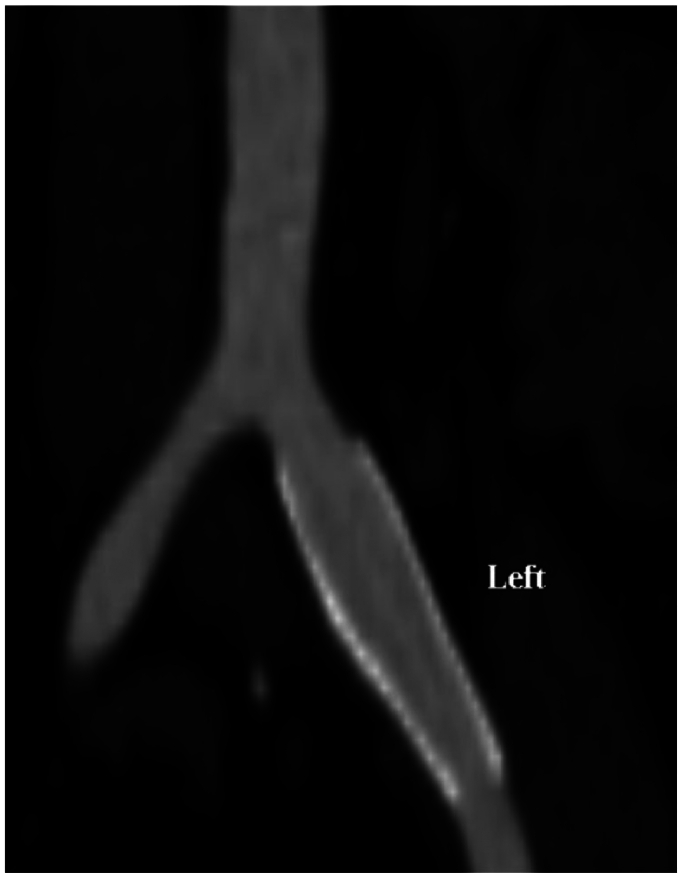
Computed tomography angiography (CTA) demonstrating successful iliac aneurysm repair with a stent graft by an outside hospital.

A 62-year-old woman presented with a left CIA aneurysm in the setting of VEDS. Her family includes siblings with VEDS, one of whom experienced a ruptured abdominal aortic aneurysm. She also has a history of hypertension, controlled by two antihypertensive medications. She underwent left CIA stent placement (with coverage of the origin of the left internal iliac artery [IIA]) at an outside hospital for a ruptured left IIA aneurysm ∼15 years previously. The stent measured ∼14 mm using our measurements and was deployed percutaneously. After initial treatment, she had regression of her iliac aneurysm (Fig 1). Subsequent imaging, however, several years later demonstrated the development of a new endoleak with expansion of the iliac artery. She did not develop any other aneurysms or dissections in the interim. She was referred with a now enlarging CIA aneurysm from a suspected type II endoleak. She has a history of colonic rupture requiring subtotal colectomy and diversion. She also had an intraductal papillary mucinous neoplasm requiring pancreaticoduodenectomy, followed by completion pancreatectomy and splenectomy.

**Fig 2:**
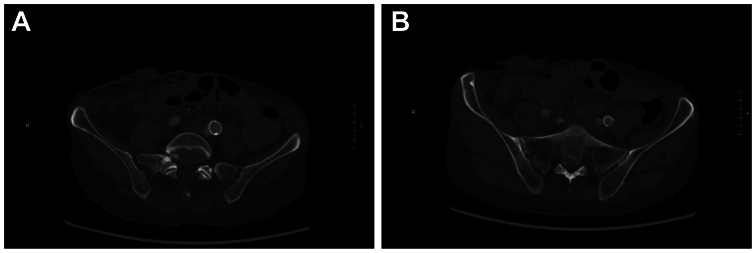
Computed tomography angiography (CTA) demonstrating an endoleak around the iliac aneurysm sac (**A**) and patent bilateral internal iliac arteries (IIAs; **B**).

She underwent a computed tomography angiography (CTA) scan that demonstrated an endoleak (Fig 2, *A*) in the CIA aneurysm sac and patent bilateral IIAs (Fig 2, *B*). The CIA aneurysm now measured 3.3 cm, which was 2.7 cm 2 years prior. At this point, either continued surveillance or surgical repair could be offered to the patient. Given her connective tissue disorder and history of prior rupture, we elected to offer the patient surgical intervention.

In regard to repair of her CIA aneurysm, both open and endovascular options were discussed with the patient. Open repair would require ligation of the IIA, which would be difficult given her extensive surgical history. Therefore, an endovascular option was the preferred treatment modality in this situation. However, embolization of the IIA would be difficult given that no direct access was available because the orifice was covered by the stent. The three endovascular options to obtain access to the IIA were as follows: (1) obtain access along the side of the stent graft, which has some increased risk of rupture and dissection given her connective tissue disease; (2) transgraft embolization; or (3) embolize the IIA via a transgluteal approach. We elected for the transgluteal approach, because we find this approach simpler and easier to perform.

**Fig 3:**
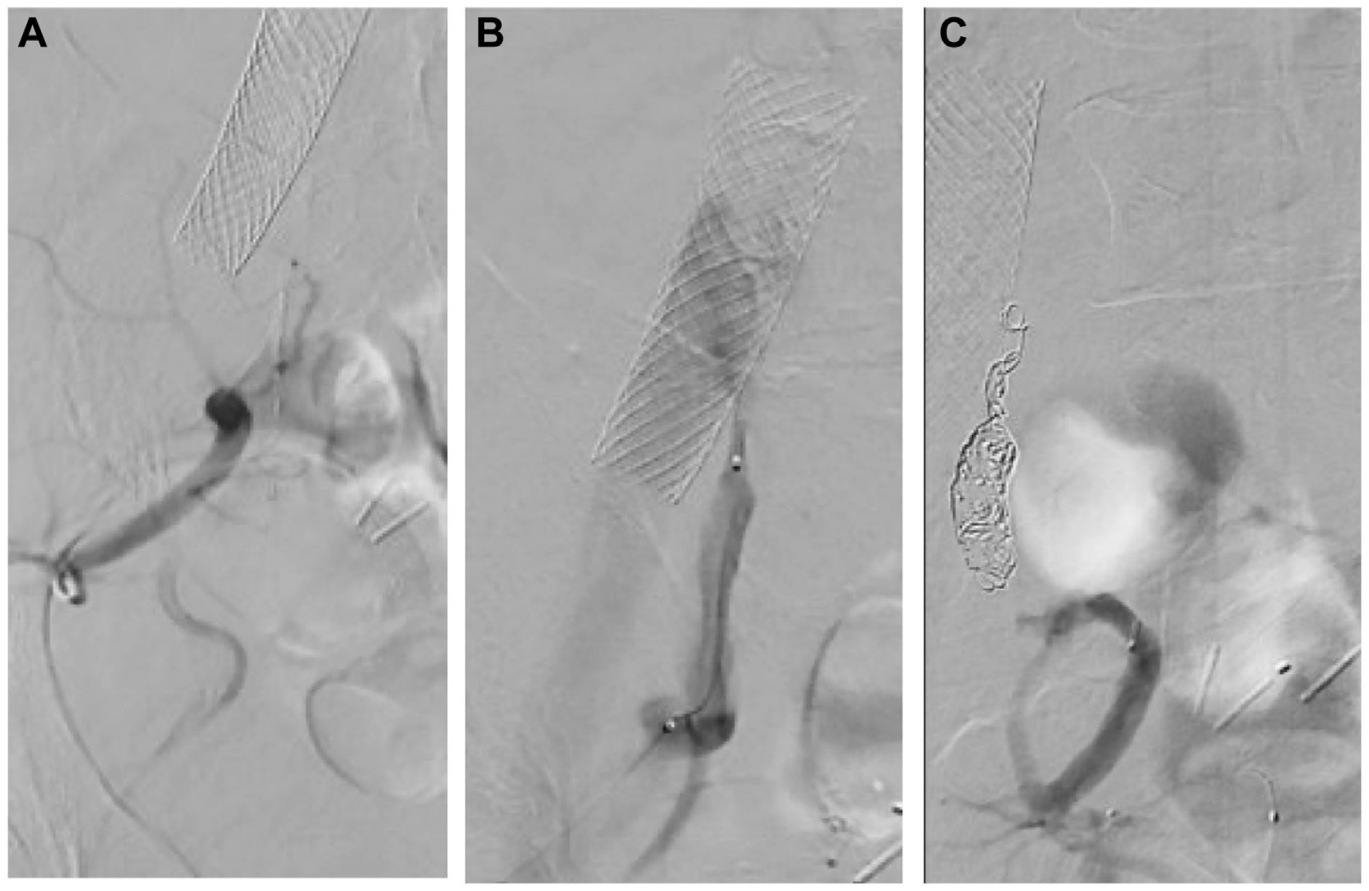
Left internal iliac artery (IIA) access and embolization via a transgluteal approach. **A,** Angiogram demonstrating the catheter within the internal iliac artery (IIA). **B,** Digital subtraction angiography demonstrating both a type Ib and a type II endoleak. **C,** Successful coiling of the IIA.

The patient was brought to the operating room and placed prone on the table. Fusion technology was used to fuse the images from the CTA with fluoroscopy to assist with obtaining access to the left gluteal branch (Fig 3, *A*). Once this vessel was accessed, a Cope Mandril Wire Guide (Cook Medical) and microcatheter system were advanced into the iliac system and to the ostium of the IIA. The Cope Mandril Wire Guide was then removed and replaced with a V18 wire (Boston Scientific) and a 0.018-in. Quick-Cross catheter (Philips) was advanced. An angiogram was performed, which demonstrated a type Ib endoleak and a type II endoleak (Fig 3, *B*). Multiple Tornado embolization coils (Cook Medical) were then packed into the IIA, and retrograde flow into the aneurysm sac was occluded (Fig 3, *C*). The catheter was then slowly retracted out of the gluteal artery and 1:1 *N*-butyl cyanoacrylate (Johnson & Johnson) with ethiodized oil was placed through the catheter near the arterial puncture site to achieve hemostasis.

**Fig 4:**
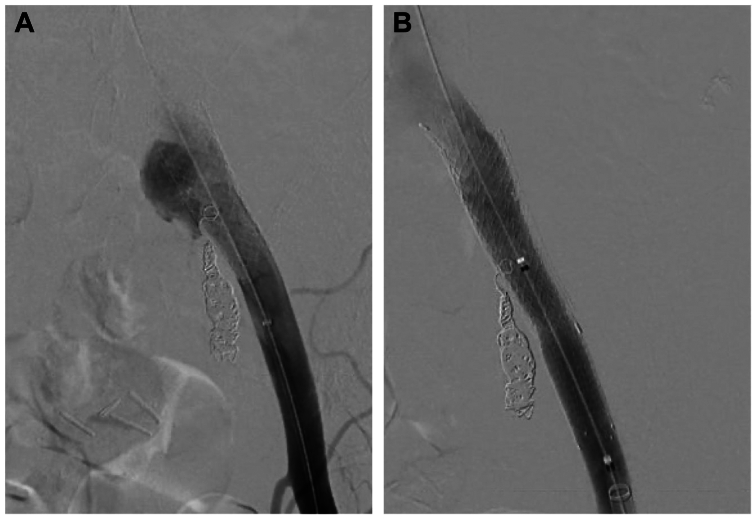
Angiography demonstrating a type Ib endoleak (**A**) and successful treatment with a stent graft (**B**).

On follow-up, the patient had a repeat CTA, which showed a persistent endoleak in the aneurysm sac. Thus, we believed a type Ib endoleak was likely, given the intraoperative findings. The patient was returned to the operating room. A left femoral artery cutdown was performed, and a retrograde angiogram was performed, which again demonstrated a type Ib endoleak. However, a type Ia endoleak was not appreciated (Fig 4, *A*). Nonetheless, there was ∼1 cm of iliac artery proximal to the iliac stent before the bifurcation. Therefore, it was decided that the entire iliac system would be relined with an endograft. A Gore iliac limb (16 mm proximally, 12 mm distally, 7 cm long; W.L Gore & Associates) was deployed into the left iliac system. An additional 13 × 5-mm Viabahn stent was placed as a distal extension. All endografts were sized as close to 1:1 (vessel/endograft) as possible to avoid traumatic vessel injury. Repeat contrast injection revealed this to be widely patent with no endoleak (Fig 4, *B*). The common femoral arteriotomy was repaired via pledgeted sutures. A follow-up CTA scan 1 month later demonstrated no evidence of an endoleak.

## Discussion

VEDS is a difficult entity to treat due to the fragility of the vascular beds.^5^ The traditional recommendation when treating vascular complications of VEDS was to prioritize a conservative approach, with surgical intervention reserved for life-threatening conditions.^6^ However, there will be times when intervention is indicated. The spectrum of disease is broad in VEDS, which can make decision-making in this population very difficult. Some patients can experience excessive tissue fragility; however, others will be able to tolerate clamping and reconstruction without significant problems.^7^

The use of stent grafts in patients with connective tissue disorders is typically not recommended due to the mismatch between the device radial force and the tensile strength of the vessel.^8^^,^^9^ Early experiences using endovascular treatment modalities in patients with connective tissue disease demonstrated extremely high complication rates with associated mortality.^10^ However, improvements in endovascular technology, in conjunction with a better understanding of connective tissue disorders, have led to the wider application of endovascular surgery to patients with VEDS.^7^ Experience with aortic and iliac stent grafting in VEDS patients is limited. Small retrospective series demonstrate that endovascular management of thoracic, thoracoabdominal, and complex abdominal aortic aneurysms is safe and feasible in patients with connective tissue disorders but is associated with device- and aorta-related complications.^11^, ^12^, ^13^ Endovascular treatment of isolated iliac aneurysms is even more rare in this patient population. Shalhub and Byers^14^ recently described a case using an iliac branch device, followed by endovascular aortic repair, in a patient with bilateral CIA aneurysms with excellent technical success. However, the long-term results for iliac aneurysms treated via an endovascular approach are still unknown.

Endoleaks from IIAs are difficult to manage, especially when the orifice is covered by an endograft. For our patient, we performed transgluteal embolization of the IIA to treat the associated type II endoleak, leading to sac expansion. Transgluteal embolization of the IIA has been previously described to treat aneurysms and endoleaks.^15^^,^^16^ However, its use in patients with VEDS has not been previously described. Our report demonstrates that this method can be safely used in VEDS patients to coil the IIA.

## Disclosures

None.
